# Comprehensive Transcriptomic Profiling Reveals Rotavirus-Induced Alterations in Both Coding and Long Non-Coding RNA Expression in MA104 Cells

**DOI:** 10.3390/v18010129

**Published:** 2026-01-20

**Authors:** Xiaopeng Song, Yanwei Wu, Xiaocai Yin, Xiaoqing Hu, Jinyuan Wu, Xiangjing Kuang, Rong Chen, Xiaochen Lin, Jun Ye, Guangming Zhang, Maosheng Sun, Yan Zhou, Hongjun Li

**Affiliations:** Yunnan Key Laboratory of Vaccine Research and Development on Severe Infectious Disease, Institute of Medical Biology, Chinese Academy of Medical Science & Peking Union Medical College, Kunming 650118, China; igtheshy131@gmail.com (X.S.);

**Keywords:** rotavirus, infection, lncRNA, mRNA, RNA-sequence, profile

## Abstract

Rotavirus (RV) is the primary cause of severe gastroenteritis in young children, yet the long noncoding RNA (lncRNA) regulatory landscape governing the host response remains largely unmapped. To address this gap, the present study performed an integrated transcriptomic analysis of mRNA and lncRNA expression profiles in RV-infected MA104 cells at 24 h post-infection. Deep sequencing identified 11,919 high-confidence lncRNAs, revealing a massive transcriptional shift: 3651 mRNAs and 4655 lncRNAs were differentially expressed, with both populations predominantly upregulated. Functional enrichment analysis confirmed the strong activation of key innate immunity pathways, including the RIG-I-like receptor, Toll-like receptor, and TNF signaling pathways. Conversely, fundamental metabolic pathways were found to be suppressed. Crucially, the analysis of lncRNA targets highlighted their involvement in coordinating the host antiviral defense, particularly through transregulation. Experimental validation confirmed the significant upregulation of key immune-related mRNAs (OASL and C3) as well as two novel lncRNAs (lncRNA-6479 and lncRNA-4290) by qRT-PCR. The significant upregulation of OASL and C3 was validated at the protein level, confirming the biological relevance of the transcriptomic data. This study provides a foundational, genome-wide resource, identifying novel lncRNA targets for future mechanistic investigation into host–RV interactions.

## 1. Introduction

Rotavirus (RV) remains a formidable global health threat, particularly as the leading cause of severe dehydrating gastroenteritis in infants and young children worldwide, despite the implementation of vaccination programs [1]. RV infection is characterized by the destruction of intestinal enterocytes, malabsorption, and significant fluid and electrolyte loss [2]. While the structural and non-structural proteins of RV, such as NSP4 and VP3, are well-known to play critical roles in viral replication, cell cycle modulation, and host innate immune subversion, the comprehensive picture of the host transcriptional response to RV infection specifically involving noncoding regulatory elements is still incomplete [3,4,5]. Previous studies have detailed how RV triggers potent innate immune responses, primarily activating Pattern Recognition Receptors (PRRs) like the Toll-like receptors (TLRs) and RIG-I-like receptors (RLRs) upon sensing viral double-stranded RNAs (dsRNAs) [6,7]. These pathways ultimately lead to the production of Type I interferons (IFNs) and proinflammatory cytokines, initiating an antiviral state [8]. However, the precise mechanisms that fine-tune these massive transcriptional cascades and maintain the balance between immune defense and immunopathology remain largely unexplored. Understanding these regulatory layers is crucial for developing targeted host-directed therapies.

In recent years, long noncoding RNAs (lncRNAs), defined as transcripts longer than 200 nucleotides with limited or no protein-coding potential, have emerged as pivotal regulators in various biological processes, including viral infections [9,10]. LncRNAs operate through diverse mechanisms, such as acting as molecular scaffolds, decoys, microRNA (miRNA) sponges, or modulating chromatin structure, thereby influencing the expression of neighboring (cis) or distant (trans) protein-coding genes [11,12]. Evidence suggests that viruses often exploit or are countered by lncRNA networks to control replication and evade host surveillance [13,14,15,16]. Indeed, while the interplay between RV and host mRNAs or miRNAs has been characterized in several studies, the regulatory landscape of lncRNAs remains largely unmapped [17,18]. Even more advanced, single-cell lncRNA transcriptomes have been successfully mapped in rhesus macaque models for Ebola virus, and single-cell RNA sequencing has recently revealed cell-type specific epithelial repair in rotavirus-infected intestinal epithelium [19,20]. These studies still do not provide a comprehensive, bulk-level map of the overall host lncRNA and mRNA transcriptional changes that orchestrate the global immune response. Furthermore, only a handful of studies have successfully identified and characterized specific lncRNA mechanisms in RV infection, notably lnc-DARVR regulating RV replication via the complement C3 pathway, and lncRNA SLC7A11-AS1 promoting ferroptosis to facilitate virus infection [21,22]. Due to the extreme scarcity of data, a comprehensive, genome-wide understanding of the overall lncRNA transcriptome dynamics and their complete regulatory networks during RV infection is severely lacking, representing a significant gap in the current understanding of RV pathogenesis.

To address this knowledge gap, the present study utilized deep sequencing technology to perform an integrated, genome-wide analysis of both mRNA and lncRNA expression profiles in MA104 cells following infection with RV. The findings revealed a massive, coordinated transcriptional shift, marked by thousands of differentially expressed mRNAs and lncRNAs. Functional analysis confirmed the activation of key antiviral pathways (RLR, TLR, TNF signaling) while simultaneously suppressing metabolic pathways. Crucially, the analysis linked trans-acting lncRNAs to the host defense response, validated by the protein upregulation of the complement component C3 and OASL. This study provides a foundational resource and identifies novel lncRNA targets for future mechanistic investigation into host–RV interactions.

## 2. Methods

### 2.1. Virus and Infection

The RV strain was originally isolated from the feces of a child in Zhaotong, Yunnan Province, and was preserved by the Institute of Medical Biology, Chinese Academy of Medical Sciences [23]. For the collection of stool samples, we obtained informed consent from the children’s parents. For the infection experiments, the cell culture medium was first removed and then washed once with phosphate-buffered saline (PBS). Subsequently, the virus inoculum containing RV at the corresponding MOI was prepared in maintenance medium and added to the cells for the specified duration.

### 2.2. Cell Culture and Maintenance

MA104 cells were cultured in Dulbecco’s modified Eagle medium (DMEM, Gibco, Grand Island, NY, USA; Cat: 11965092) supplemented with 10% fetal bovine serum (FBS, Gibco; Cat: 1921005PJ) and 1% penicillin-streptomycin antibiotics (Gibco; Cat: P6038441). Cells were maintained at 37 °C in a humidified atmosphere containing 5% CO_2_. Cells were regularly checked for Mycoplasma contamination.

### 2.3. RNA Extraction and Library Construction

After removing the cell culture supernatant, cells were washed twice with PBS. Total cellular RNA was then extracted using Trizol (ThermoFisher, Waltham, MA, USA; cat: 15596026) reagent. Library preparation was performed by ApexBio Technology (Shanghai, China). For lncRNA-Seq, 2 μg of total RNA was first subjected to ribosomal RNA depletion using the QIAseq Fast Select-rRNA HMR Kit (QIAGEN, Hilden, Germany; cat: 334376). Libraries were then generated using the VAHTS Universal V8 RNA-Seq Library Prep Kit (Vazyme, Nanjing, China; Cat: NR605-01) and verified for quality on the Agilent Bioanalyzer 4200 system. Libraries were sequenced on the Illumina Xplus platform (Paired-end 150) to generate raw reads from three independent biological replicates per group.

### 2.4. Bioinformatics Analysis

Raw paired-end reads were first quality-filtered using Fastp to remove adapter sequences and low-quality bases. The resulting clean reads were aligned to the *Macaca mulatta* reference genome using Hisat2 (v2.2.1) [24]. MA104 cells are derived from African green monkey (*Chlorocebus sabaeus*) kidney epithelium. RNA-Seq reads were aligned to the *Macaca mulatta* reference genome due to its more complete annotation. Aligned reads were then assembled with StringTie (v1.3.1) to reconstruct the final transcriptome [25]. Detailed sequencing quality and mapping metrics are provided in Supplementary Table S1. The transcript abundance for mRNAs was quantified as Transcripts Per Million (TPM). LncRNAs were identified through a stringent multi-step pipeline. Transcripts shorter than 200 bp or containing fewer than two exons were excluded, followed by the removal of sequences overlapping known coding or annotated lncRNA regions. The remaining transcripts were subjected to coding potential evaluation using the CNCI, CPC2, and Pfam databases, and only transcripts consistently predicted as noncoding were retained as high-confidence lncRNAs [26,27,28].

Differential expression analysis for all coding and noncoding transcripts was performed using the DESeq2 R package (v 1.50.2; suitable for samples with replicates) with a cut-off threshold of |log_2_(Fold Change)| ≥ 1 and adjusted *p* < 0.05 [29]. Functional enrichment analysis for the significant differentially expressed genes (DEGs) was conducted using the clusterProfiler R package, evaluating enrichment based on Gene Ontology (GO) and KEGG pathway categories [30]. Terms with *p* < 0.05 were considered statistically significant. lncRNA target prediction employed a cisregulation approach (mRNAs within 100 kb proximity) and a transregulation approach (based on strong Pearson Correlation |r| > 0.95), followed by GO/KEGG enrichment analysis of the predicted target mRNAs.

### 2.5. qRT-PCR

For quantitative validation of gene expression, reverse transcription quantitative PCR (qRT-PCR) was performed using the HiScript II One Step qRT-PCR SYBR Green Kit (Vazyme, Cat: Q222/Q221). These kits were used for the detection of both host mRNA/lncRNA and viral genomic RNA. The relative expression levels of all host target genes and lncRNAs were normalized to the expression of β-actin as the internal reference gene. Relative gene expression data were then calculated using the comparative threshold cycle (2^−ΔΔCt^) method. All primer sequences utilized in this study are detailed in Table 1.

### 2.6. RNAi and Transfection

All siRNAs targeting lncRNA C3, OASL, and lncRNA 6479 were designed and synthesized by Gene Pharma (Shanghai, China), together with the corresponding negative control siRNAs. The sequences of all RNA oligonucleotides are listed in Supplementary Table S2. Transfections were performed using Lipofectamine™ 3000 Reagent (Thermo Fisher, L3000008, Waltham, MA, USA) according to the manufacturer’s instructions.

### 2.7. Immunofluorescence Assay

Infected MA104 cells grown on coverslips were first fixed using 4% paraformaldehyde solution containing 0.2% Triton-X 100 for 30 min. Following washes, the fixed cells were incubated with a purified goat anti-RV primary antibody (Institute of Medical Biology) and C3 antibody (Proteintech, Chicago, IL, USA; Cat: 21337-1-AP). This was followed by incubation with the secondary antibody. Cell nuclei were counterstained with DAPI (Solarbio, Beijing, China; Cat: C0065). Fluorescent images were captured using an inverted fluorescence microscope.

### 2.8. Western Blotting

Cells were harvested after washing with PBS, and total cellular protein was extracted on ice using RIPA lysis buffer (Beyotime, Shanghai, China; Cat: P0013B). Protein concentrations in the lysates were determined using the BCA Protein Assay Kit (Beyotime, Cat: P0009). Equal amounts of protein were resolved by SDS-PAGE and transferred to PVDF membranes. Membranes were blocked and subsequently incubated with specific primary antibodies of OASL (Abclonal, Woburn, MA, USA; Cat: A24529), C3 (Proteintech, Cat: 21337-1-AP), NSP3 (Institute of Medical Biology), VP7 (Institute of Medical Biology) and GAPDH (Abclonal, Cat:19056), followed by HRP-conjugated secondary antibodies. Blots were visualized using an enhanced chemiluminescence detection system.

### 2.9. Statistical Analysis

All quantitative data are expressed as the mean ± the standard error (SE) derived from at least three independent biological replicates. Statistical comparisons between two experimental groups were performed using the two-tailed Student’s *t*-test. For multiple group comparisons, one-way analysis of variance (ANOVA) was applied, followed by Duncan’s multiple comparison test for post hoc analysis. Differences were considered statistically significant when the *p*-value was less than 0.05 (*p* < 0.05).

## 3. Results

### 3.1. Establishment and Characterization of Rotavirus Infection in MA104 Cells

**Figure 1:**
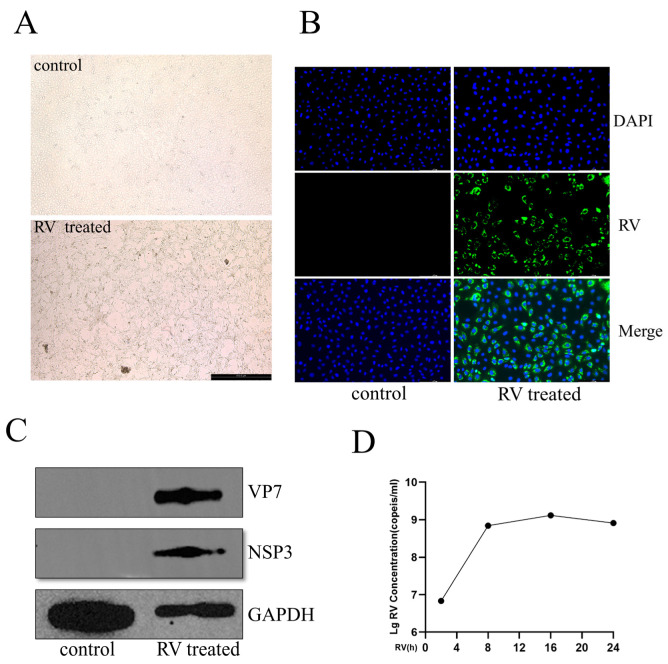
Characterization of RV Infection Kinetics and Pathology in MA104 Cells. (**A**) Phase-contrast images showing the cytopathic effects (CPE) induced by Rotavirus (RV) infection (MOI of 0.1) in MA104 cells at 24 hpi. (**B**) Immunofluorescence (IF) analysis of RV antigen expression (green) in MA104 cells at 24 hpi (MOI of 0.1). Cell nuclei are counterstained with DAPI (blue). (**C**) Western blot analysis of protein lysates from MA104 cells at 24 hpi (MOI of 0.1). (**D**) Time course of RV NSP3 RNA replication kinetics (MOI of 0.1). Data are presented from three independent experiments.

To establish a robust in vitro model for subsequent transcriptomic analysis, MA104 cells were infected with RV at an MOI of 0.1, and infection kinetics were characterized. At 24 h post-infection (hpi), phase-contrast microscopy (Figure 1A) showed evident cytopathic effects (CPE), including cellular rounding and detachment, in RV-infected cells, while the DMEM-treated controls maintained their healthy morphology. Successful viral replication and expression were verified at 24 hpi. Immunofluorescence (IF) microscopy revealed widespread cytoplasmic RV (MOI of 0.1) antigen distribution (Figure 1B). Consistent with this, Western blot analysis confirmed the robust accumulation of viral proteins NSP3 and VP7 (Figure 1C). The temporal kinetics of viral RNA replication were assessed by qRT-PCR quantification of RV NSP3 RNA levels across 2, 8, 16, and 24 hpi. NSP3 RNA abundance increased significantly and continuously from 2 hpi up to 16 hpi (MOI of 0.1), suggesting active viral replication (Figure 1D). Crucially, the viral RNA level reached a plateau between 16 hpi and 24 hpi. Based on these findings, the 24 hpi time point, representing the late stage of the viral replication cycle and maximal accumulation, was selected for subsequent deep sequencing analysis.

### 3.2. Identification and Characterization of Host Coding and Non-Coding Transcripts

**Figure 2:**
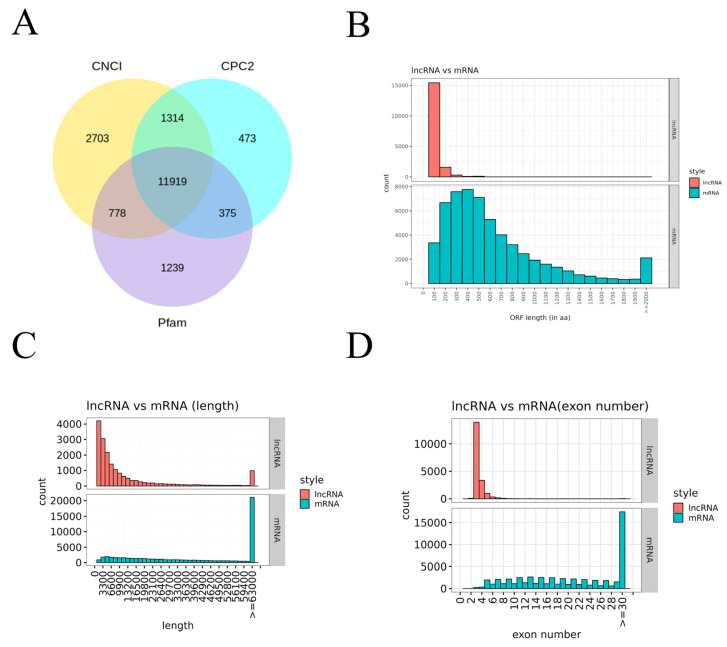
Genome-wide Identification and Structural Characterization of lncRNAs in MA104 Cells: (**A**) Venn diagram illustrating the stringent three-tool intersection strategy used for identifying lncRNAs. (**B**) Comparison of ORF length distribution between the identified lncRNAs and mRNAs. (**C**) Comparison of transcript length distribution between lncRNAs and mRNAs. (**D**) Comparison of exon number distribution between lncRNAs and mRNAs.

To comprehensively analyze the host response to RV infection, deep sequencing was performed on total RNA from RV-infected (24 hpi) and control MA104 cells. Reads were assembled to identify both known and novel transcripts, focusing on lncRNAs. High-confidence lncRNAs were identified using a stringent three-tool intersection approach (Figure 2A). After excluding coding transcripts via CNCI, CPC2, and Pfam analysis, a final set of 11,919 lncRNAs was successfully identified. Structural characteristics of these lncRNAs were then compared against mRNAs (Figure 2B–D). Analysis of the Open Reading Frame (ORF) length showed that while the majority of lncRNAs clustered around 100 bp (approx. 15,000 transcripts), mRNA ORFs exhibited a much wider range, from 200 bp to over 2000 bp (Figure 2B). This difference extended to transcript length (Figure 2C): the most abundant lncRNAs (approx. 4000 transcripts) were clustered around 3300 bp, whereas the highest number of mRNAs (approx. 20,000 transcripts) were longer than 6300 bp. Furthermore, lncRNAs contained significantly fewer exons (Figure 2D); over 10,000 lncRNAs were characterized by having only four exons, while the most frequently observed mRNAs contained 30 exons (over 15,000 transcripts). These structural differences collectively confirm that the identified lncRNAs exhibit the typical features of noncoding transcripts.

### 3.3. Differential Expression Analysis of mRNAs and lncRNAs in Response to RV Infection

**Figure 3:**
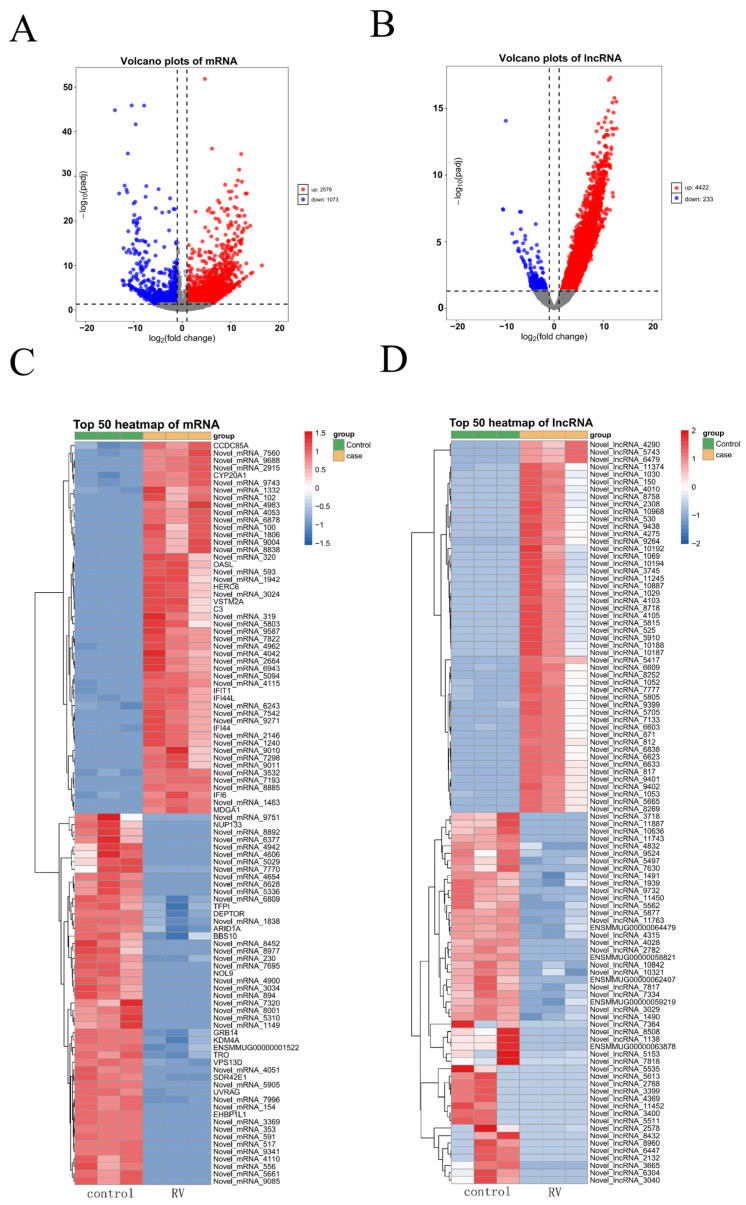
Identification of Differentially Expressed mRNAs and lncRNAs in RV-Infected MA104 Cells: (**A**) Volcano plot illustrating the differentially expressed mRNAs between RV-infected (24 hpi) and control MA104 cells. (**B**) Volcano plot illustrating the differentially expressed lncRNAs between RV-infected and control MA104 cells. (**C**) Heatmap representation of the top 50 most significantly up- and downregulated mRNAs in RV-infected MA104 cells. (**D**) Heatmap representation of the top 50 most significantly up- and downregulated lncRNAs.

Following the characterization of host transcripts, the differential expression analysis was performed to identify mRNAs and lncRNAs significantly altered by RV infection at 24 hpi. Transcripts with a |log2(Fold Change)| ≥ 1 and a −log10(adjusted *p*-value) ≥ 1.3 (corresponding to adjusted *p*-value ≤ 0.05) were considered differentially expressed. The volcano plot for mRNAs (Figure 3A) revealed a substantial number of transcripts regulated by RV. Specifically, 2578 mRNAs were significantly upregulated, and 1073 mRNAs were significantly downregulated in infected cells compared to control cells. This demonstrated a strong host transcriptional response to the infection. A similar analysis for lncRNAs showed an even more pronounced shift toward upregulation (Figure 3B). A total of 4422 lncRNAs were significantly upregulated, while only 233 lncRNAs were significantly downregulated. The magnitude of lncRNA upregulation, combined with the higher number of upregulated compared to downregulated transcripts, suggests that lncRNAs play a critical role in the host’s response, potentially favoring viral replication or host defense mechanisms. To highlight the most robust transcriptional changes, the top 50 significantly up- and downregulated mRNAs were examined (Figure 3C). Several genes previously associated with antiviral defense and inflammation, such as OASL, C3, IFIT1, and IFI6, were among the most highly induced [31,32,33]. Other highly upregulated genes included CCDC85A and CYP20A1. Conversely, genes like EHBP1L1, UVRAG1, and TRO exhibited significant downregulation, suggesting the suppression of certain cellular pathways. The top 50 most differentially expressed lncRNAs were also profiled (Figure 3D). Strikingly, the majority of the most significantly upregulated lncRNAs were newly identified in this study. In the downregulated group, only five lncRNAs had previous annotations, with the rest being novel transcripts, highlighting the significant discovery of novel regulatory elements in the host response.

### 3.4. Functional Enrichment Analysis Reveals Activation of Antiviral and Inflammatory Pathways

**Figure 4:**
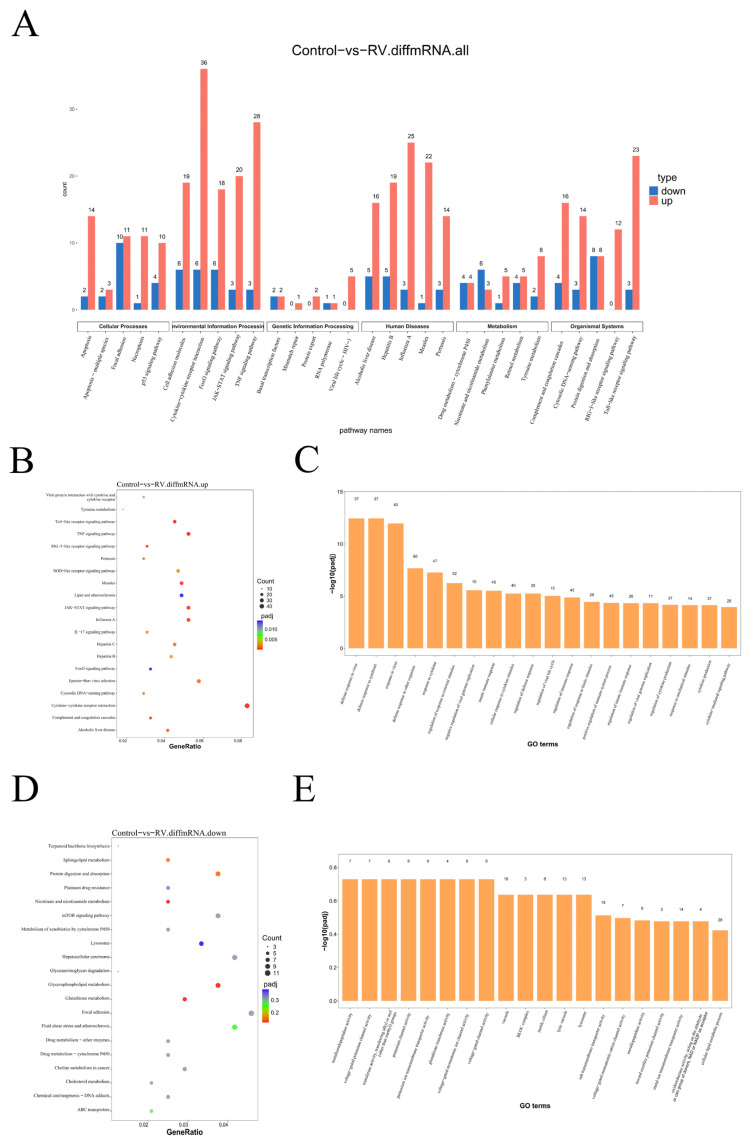
Functional Characterization of Differentially Expressed mRNAs in RV-Infected MA104 Cells: (**A**) KEGG pathway enrichment analysis of all DEmRNAs. (**B**) KEGG pathway enrichment analysis of significantly upregulated mRNAs. (**C**) GO enrichment analysis of significantly upregulated mRNAs. (**D**) KEGG pathway enrichment analysis of significantly downregulated mRNAs. (**E**) GO enrichment analysis of significantly downregulated mRNAs.

To functionally interpret the large number of differentially expressed mRNAs (DEmRNAs), we performed KEGG pathway and GO enrichment analyses. KEGG pathway enrichment of all DEmRNAs (Figure 4A) indicated that the host response was primarily focused on immune and viral-related processes. Notably, the most enriched pathway was “Cytokine–cytokine receptor interaction,” which included 36 upregulated and 6 downregulated mRNAs. This was closely followed by pathways such as the “TNF signaling pathway” and “Influenza A”, underscoring a strong inflammatory and infectious response signature. Within the “Organismal Systems” category, several innate immune signaling pathways, including the “Complement and coagulation cascades”, “RIG-I-like receptor signaling pathway”, and “Toll-like receptor signaling pathway” were also significantly enriched with DEmRNAs. Focusing specifically on the upregulated mRNAs (Figure 4B), the analysis highlighted core antiviral and inflammatory signaling modules. The top enriched pathways included “Viral protein interaction with cytokine and cytokine receptor”, “Toll-like receptor signaling pathway”, “RIG-I-like receptor signaling pathway”, and “NOD-like receptor signaling pathway”, confirming the massive activation of innate immunity and inflammation following RV infection. GO enrichment analysis of the upregulated mRNAs (Figure 4C) further supported the observed immune response. The major enriched biological process terms were related to “Defense response to virus”, “Response to virus”, and the regulation of the immune system and immune effector processes. Conversely, KEGG analysis of the downregulated mRNAs (Figure 4D) suggested the suppression of fundamental cellular metabolic and growth pathways. Key enriched pathways included “Protein digestion and absorption”, the “mTOR signaling pathway”, and “Lysosome”, indicating that RV infection may globally inhibit host anabolic processes to favor viral replication. The GO analysis of the downregulated mRNAs (Figure 4E) showed enrichment in terms like “metalloendopeptidase activity”, though this term was only associated with 7 genes, suggesting a more targeted or minor suppression effect on specific enzymatic functions compared to the broad metabolic suppression.

### 3.5. Functional Characterization of Differentially Expressed lncRNAs Target Genes

**Figure 5:**
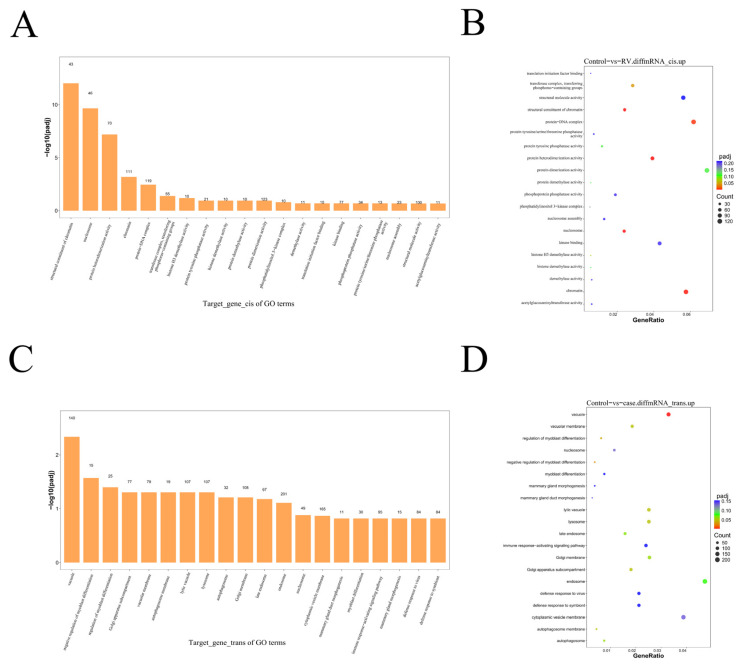
Functional Enrichment Analysis of Target mRNAs Regulated by Differentially Expressed lncRNAs. (**A**) GO enrichment analysis of upregulated mRNAs regulated in cis by DElncRNAs. (**B**) KEGG pathway enrichment analysis of upregulated mRNAs regulated in cis by DElncRNAs. (**C**) GO enrichment analysis of upregulated mRNAs regulated in trans by DElncRNAs. (**D**) KEGG pathway enrichment analysis of upregulated mRNAs regulated in trans by DElncRNAs.

To investigate the potential regulatory roles of differentially expressed lncRNAs (DElncRNAs), their target mRNAs were identified, and subsequent functional enrichment analyses were performed. GO enrichment analysis of the upregulated mRNAs predicted to be regulated in cis (Figure 5A) highlighted terms related to chromatin structure and organization. Specifically, “structural constituent of chromatin” was enriched with 43 genes, and “nucleosome” was enriched with 46 genes, suggesting that lncRNA-mediated cisregulation may play a role in altering the host cell’s genetic architecture. KEGG pathway analysis of the cisregulated upregulated mRNAs (Figure 5B) showed significant enrichment in “Viral carcinogenesis”, indicating a potential link between the RV-induced lncRNA regulation and pathways associated with cellular transformation or persistent viral mechanisms. Analysis of mRNAs predicted to be regulated in trans by DElncRNAs showed a different functional profile. GO analysis of the transregulated upregulated mRNAs (Figure 5C) identified “vacuole” as the term with the highest number of enriched genes. Crucially, the term “defense response to virus” was also significantly enriched, mapping to 84 target genes, suggesting that lncRNA transregulation contributes substantially to the overall host antiviral defense, highlighting the pervasive nature of lncRNA involvement in coordinating the innate immune response. KEGG pathway analysis of the transregulated upregulated mRNAs (Figure 5D) revealed significant enrichment in major host signaling pathways, including the “TNF signaling pathway”, “mTOR signaling pathway”, and “MAPK signaling pathway”, which are central to inflammation, proliferation, and stress response. The prominent enrichment of these pathways underscores the multifaceted role of trans-acting lncRNAs in modulating key host defenses and cellular survival mechanisms following RV infection.

### 3.6. Validation of Key Transcriptomic and Proteomic Changes Following Rotavirus Infection

**Figure 6:**
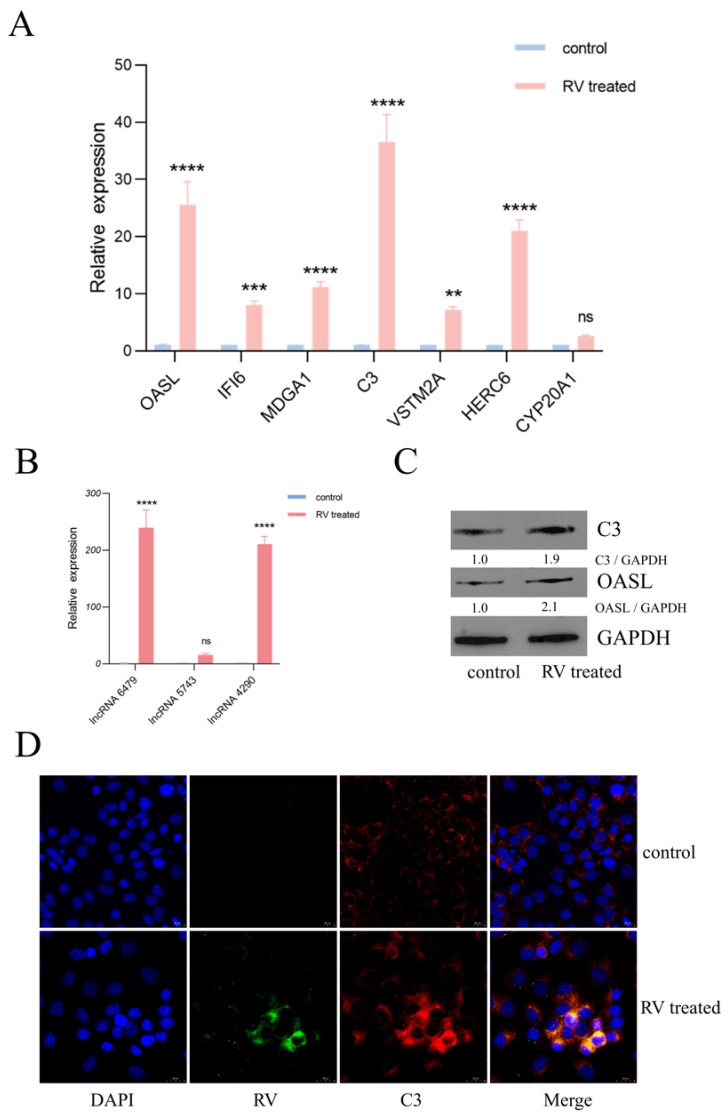
Validation of Key Transcriptomic and Proteomic Changes Following Rotavirus Infection: (**A**) qRT-PCR Validation of selected differentially expressed mRNAs in MA104 cells infected with RV (0.1 MOI, 24 hpi). (**B**) qRT-PCR validation of three novel lncRNAs in RV-infected MA104 cells (0.1 MOI, 24 hpi). (**C**) Western blot analysis of C3 and OASL protein levels in MA104 cells at 24 hpi (0.1 MOI RV). (**D**) Immunofluorescence colocalization of C3 protein and RV antigen at 24 hpi. Data are presented from three independent experiments. (ns, not significant; ** *p* < 0.01, *** *p* < 0.001, **** *p* < 0.0001).

The expression levels of seven significantly upregulated mRNAs (OASL, IFI6, MDGA1, C3, VSTM2A, HERC6, and CYP20A1) were quantified by qRT-PCR in MA104 cells infected with RV (0.1 MOI, 24 hpi) compared to the DMEM control (Figure 6A). Consistent with the sequencing data, six of the seven selected genes (OASL, IFI6, MDGA1, C3, VSTM2A, and HERC6) were confirmed to be significantly upregulated in RV-infected MA104 cells. However, the expression of CYP20A1 did not show a statistically significant change in the qRT-PCR assay. Furthermore, the expression levels of three novel lncRNAs (lncRNA-6479, lncRNA-5743, and lncRNA-4290) were validated by qRT-PCR (Figure 6B), and the DEmRNAs and DElncRNAs identified in HT-29 and Caco-2 cells are shown in Supplementary Figures S1 and S2. The results demonstrated that lncRNA-6479 and lncRNA-4290 were significantly upregulated following RV infection, confirming the RNA-Seq data. In contrast, lncRNA-5743 showed no discernible change in expression. To confirm the functional relevance of the observed mRNA changes, the protein levels of two highly upregulated immune-related genes, C3 and OASL, were assessed by Western blot at 24 hpi (Figure 6C). Densitometric analysis revealed a 1.9-fold upregulation for the C3 protein and a 2.1-fold upregulation for the OASL protein in RV-infected cells compared to control cells, thereby validating the transcriptional findings at the translational level. Finally, the subcellular localization of the C3 protein and the RV antigen was investigated via IF colocalization at 24 hpi (Figure 6D). The images confirmed that C3 and RV colocalized following 24 h of infection, suggesting a direct or indirect interaction or coordinated presence within the same cellular compartment, potentially related to the active viral replication site. Following the knockdown of OASL, C3, lncRNA6479, and lncRNA4290 via siRNA interference and subsequent infection with RV, the results showed that the silencing of OASL, C3, and lncRNA4290 significantly promoted RV replication. Conversely, the interference of lncRNA6479 exhibited an inhibitory trend on RV replication (Supplementary Figures S3–S5).

## 4. Discussion

To accurately characterize the host response to Rotavirus (RV) infection, a robust in vitro model in MA104 cells was successfully established. The observed CPE, marked by cellular rounding and detachment at 24 hpi with a low multiplicity of infection (MOI) of 0.1 (Figure 1A,B), is consistent with the typical lytic nature of RV replication [21]. The successful detection of viral antigens (VP7 and NSP3) via IF by Western blotting, coupled with the sharp rise in NSP3 RNA levels peaking between 16 hpi and 24 hpi (Figure 1C,D), confirms active viral propagation and progression to the late stage of the replication cycle [7]. The selection of the 24 hpi time point for deep sequencing was strategically chosen to capture the full spectrum of host gene expression changes at a time when viral synthesis is highly active but has achieved maximal accumulation, providing insights into the sustained host response.

A comprehensive analysis of the deep sequencing data led to the robust identification of 11,919 lncRNAs (Figure 2A), successfully distinguishing them from protein-coding mRNAs based on established criteria (CNCI, CPC2, and Pfam). Structural comparisons (Figure 2B–D) confirmed that lncRNAs in MA104 cells, consistent with mammalian trends, possess significantly shorter ORFs, smaller overall transcript lengths, and fewer exons compared to mRNAs [34,35]. These structural differences underpin their functional distinction and necessity for separate analysis in regulatory networks.

Specifically, 2578 mRNAs were upregulated and 1073 were downregulated. The differential expression pattern of lncRNAs was highly skewed, with 4422 lncRNAs upregulated versus only 233 downregulated (Figure 3A,B), suggesting that the primary role of host lncRNAs in response to RV is overwhelmingly one of the activation and promotion of specific regulatory pathways rather than suppression. This dramatic upregulation highlights a massive transcriptional response aimed at mounting an effective cellular defense. The top 50 differentially expressed mRNAs included several genes well-known for their roles in antiviral defense and innate immunity (Figure 3C), such as OASL, IFIT1, IFI6, and C3 [31,32,33,36]. The strong upregulation of these genes is a direct indication of the host cell entering a robust interferon stimulated state, a critical component of the anti-rotaviral response. Conversely, downregulated genes like EHBP1L1 and UVRAG suggest that the virus employs specific counter-strategies to subvert key host machinery. The suppression of EHBP1L1, which is known to stabilize JAK1 (a central kinase in the IFN–JAK–STAT signaling pathway), may represent a viral attempt to dampen the host’s potent antiviral response [37]. Similarly, the reduced expression of UVRAG, a critical mediator of autophagosome maturation and endocytic trafficking, may indicate a strategy to inhibit essential cellular defensive processes, thus facilitating viral replication and accumulation [38].

KEGG pathway and GO function analysis of the differentially expressed mRNAs provided deep functional context. The most highly enriched pathways among upregulated mRNAs were fundamentally rooted in antiviral defense (Figure 4C). The enrichment of the cytokine–cytokine receptor interaction pathway, involving a significant number of 36 upregulated genes (Figure 4B), directly points to a viral-induced inflammatory and communication state within the cell. Furthermore, the strong enrichment of the TNF signaling pathway, RLR signaling pathway, and TLR signaling pathway confirms that RV infection in MA104 cells rapidly and broadly activates the core pathogen recognition and immune response cascades (Figure 4B). These pathways are central to the dsRNAs and the subsequent initiation of the Type I IFN response [39]. The GO analysis (Figure 4C) supported this by showing dominant enrichment in terms of “defense response to virus”, “response to virus”, and “regulation of immune system process”. These findings collectively confirm that the infected MA104 cells mount a powerful, systemic innate immune defense, a characteristic common to RV infection models. In stark contrast, downregulated mRNAs were enriched in pathways associated with fundamental cellular processes, primarily protein digestion and absorption, mTOR signaling pathway, and lysosome function (Figure 4D). This pattern suggests a deliberate or indirect viral strategy to redirect cellular resources away from growth, metabolism, and protein turnover, likely to free up machinery for virion production and potentially suppress pro-apoptotic signals until viral egress is complete. The suppression of the mTOR pathway is particularly noteworthy (Figure 4E), as mTOR is a master regulator of cell growth that many viruses manipulate to their advantage [40].

The integration of lncRNA and mRNA differential expression data provided insight into potential regulatory mechanisms. The functional enrichment analysis of target mRNAs regulated by lncRNAs revealed distinct functional roles for cis- and transregulation. mRNAs targeted by cis-acting lncRNAs primarily enriched for structural and organizational functions (Figure 5A,B), specifically “structural constituent of chromatin” and “nucleosome”. This suggests that lncRNAs acting locally may be involved in modulating chromatin structure and gene accessibility at or near their site of transcription, a common mechanism by which lncRNAs regulate neighboring genes [41,42]. The enrichment in “Viral carcinogenesis” may indicate that, similar to other viral models, lncRNA dysregulation could inadvertently affect cellular growth control mechanisms, even if not directly carcinogenic in this acute infection model. In contrast, the mRNAs regulated by trans-acting lncRNAs showed enrichment in more dynamic, signal-transduction pathways, including “defense response to virus”, “TNF signaling pathway”, and MAPK signaling pathway (Figure 5C,D). This finding is highly significant, as it suggests that lncRNAs acting distally (via RBP binding or other complex formation) play a major role in coordinating the large-scale, functional immune response [43]. The enrichment of 84 genes in “defense response to virus” highlights these trans-acting lncRNAs as potential master regulators connecting RV detection to downstream antiviral execution. This complexity underscores the necessity of studying lncRNAs to fully map the host–virus interaction landscape.

The results from deep sequencing were strongly validated by RT-qPCR for selected key differentially expressed mRNAs OASL IFI6, MDGA1, C3, VSTM2A, HERC6, and novel lncRNAs, novel lncRNA 6479 and 4290 (Figure 6A,B). The high concordance between the sequencing data and the qPCR-PCR results confirms the reliability of the transcriptomic analysis. Although CYP20A1 was an exception, potentially due to transient expression dynamics or post-transcriptional regulation not captured by the bulk sequencing data, the general trend of upregulation was robust. The subsequent validation of C3 and OASL protein expression via Western blot (1.9-fold and 2.1-fold increase, respectively) further cemented the biological relevance of these transcriptomic changes (Figure 6C). The demonstrated co-localization of C3 and RV antigens at 24 hpi suggests a direct involvement of the complement component C3 at the site of viral replication or assembly (Figure 6D), possibly indicating an interaction between the complement system and intracellular antiviral defense mechanisms [21].

Knockdown of OASL, C3, and lncRNA4290 significantly promotes RV replication, confirming their stable and potent antiviral functions. Notably, lncRNA4290 stands out as a novel regulatory candidate whose strong antiviral activity warrants further investigation to uncover its unique role in host defense. Conversely, the interference of lncRNA6479 appeared to inhibit RV replication, although this inhibitory effect did not reach statistical significance (Supplementary Figures S3–S5). These functional validations definitively establish the involvement of both coding and non-coding RNAs in the host immune response against RV infection.

## 5. Conclusions

In summary, this study presents the first comprehensive, genome-wide profiling of lncRNA and mRNA expression in RV-infected MA104 cells. The analysis revealed robust activation of innate immune signaling pathways, including RLR, TLR, and TNF, alongside the suppression of the mTOR metabolic pathway. Thousands of novel lncRNAs were identified, and their widespread regulatory potential was demonstrated, particularly through trans-acting mechanisms that modulate the antiviral defense response. Among these, lncRNA-6479 and lncRNA-4290 were markedly upregulated and may serve as promising candidates for further functional investigation. The protein-level validation of C3 and OASL confirmed the biological relevance of the transcriptomic findings. Collectively, this work provides a valuable resource and establishes a critical foundation for future studies aimed at elucidating how specific lncRNAs, such as lncRNA-6479 and lncRNA-4290, associated with host–RV interactions and potentially contribute to the key antiviral gene expression.

## Figures and Tables

**Table 1:** qRT-PCR primers.

	FORWARD	REVERSE
MDGA1	TGCGGCATCCCAGACAAGG	CCACCAGAGTTTCGTTCACAGAC
C3	AAGATAAGAACCGCTGGGAGGAG	TTCATTGAGCCAACGCACGAC
OASL	TACAGCACGCCAGCCTCCAG	CTCCTCCACGGTCCGCACAG
VSTM2A	ACCAAGATTAGCACAGTGAAAGTCC	TTCCTGAAGCTCCCCGTAGTTG
HERC6	ATGGCCTAGTTTCACAGATAGATTGC	TGAGTTGGCTTGCTTGTGTCAC
CYP20A1	ATCAATCTGGTGGTGGCAATGTG	GGAGGAGGGCAAAATTACTCTTCAG
CCDC85A	ACACGCCAGGCACAGTGG	GGCTCTGGGAAGATGCTCTAGG
β-actin	CAGATGTGGATCAGCAAGCAGGAG	CAGTAACAGTCCGCCTAGAAGCAC
LncRNA6479	GCATGTTCTGCTCCTGGTTCTG	ACCCTTTCGTGTGTAGGTATGTTTAC
LncRNA5743	GAGGAAGGCTAGGAGTTGGTAGAAG	TTCAGAGGCAGCAGCAGTGAG
LncRNA4290	AACCTGCTGCCCTGCTCATAG	ACTATCTACCTCTGCGACTTCCATG
NSP3	ACCATCTACACATGACCCTC	GGTCACATAACGCCCC
NSP3 Probe	ATGAGCACAATAGTTAAAAGCTAACACTGTCAA	

## Data Availability

The original contributions presented in this study are included in the article/Supplementary Materials. Further inquiries can be directed to the corresponding author(s).

## Supplementary Materials

The following supporting information can be downloaded at: https://www.mdpi.com/article/10.3390/v18010129/s1, Figure S1: qRT-PCR validation of mRNAs in HT-29 and caco-2 cells infected with RV (0.3 MOI, 24 hpi); Figure S2: qRT-PCR validation of lncRNAs in HT-29 and caco-2 cells infected with RV (0.3 MOI, 24 hpi); Figure S3: the siRNA experiment of C3 and OASL; Figure S4: the siRNA experiment of lncRNA 6479 and lncRNA4290. Figure S5: the effect of C3, OASL and lncRNA 6479 inhibition on RV replication (0.1 MOI, 16 hpi after transfection for 48 h); Table S1: sequencing quality metrics; Table S2: siRNAs.
