# Supplementary material for: Comprehensive Transcriptomic Profiling Reveals Rotavirus-Induced Alterations in Both Coding and Long Non-Coding RNA Expression in MA104 Cells
Source: Viruses. 2026 Jan 20;18(1):129. doi: 10.3390/v18010129 (PMC12846614; doi:10.3390/v18010129)
Supplement: Supplementary file 1 [file viruses-18-00129-s001.zip › viruses-4037868-supplementary.pdf]

Methods

Table S1. Sequencing quality metrics.

Sample ID	Group	Library type	Clean reads (M)	Read length (bp)	Q20 (%)	Mapping rate (%)	Expressed mRNAs	Expressed lncRNAs
Control 1	Control	Stranded (ISR)	63.93	150	98.47	93.60	19261	7279
Control 2	Control	Stranded (ISR)	66.48	150	98.45	93.86	19419	7391
Control 3	Control	Stranded (ISR)	66.15	150	98.54	94.01	19211	6844
RV 1	RV	Stranded (ISR)	359.43	150	98.66	93.24	20658	10884
RV 2	RV	Stranded (ISR)	437.00	150	98.68	96.83	19521	10151
RV 3	RV	Stranded (ISR)	91.50	150	98.52	93.63	19664	8616

Table S2. siRNAs

	Sense	Antisense
LncRNA 6479	GGUUAUUUCUAUUAAAUUATT	UAAUUUAAUAGAAAUAACCTT
LncRNA 4290	GGUCUCAGUUACUGGUUUATT	AAUAAACCAGUAACUGAGATT
OASL	GGGUCUGUUGCUAUGACAATT	UUGUCAUAGCAACAGACCCTT
C3	GGUCAACUCACCUGUAAUATT	UAUUACAGGUGAGUUGACCTT

Results

qRT-PCR validation of selected differentially expressed mRNAs in HT-29 and caco-2 cells infected with RV.

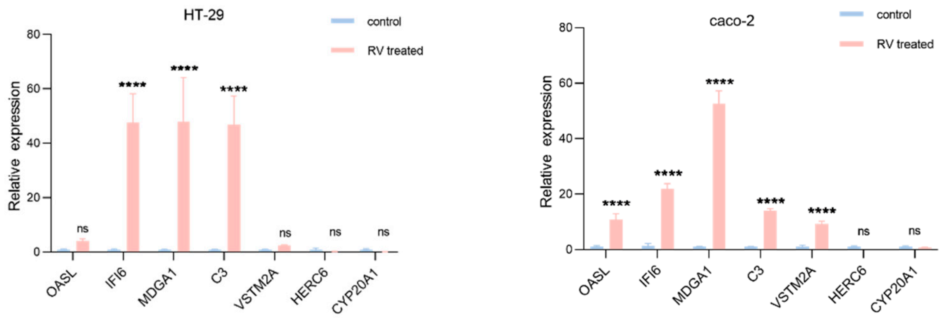

Figure S1. qRT-PCR validation of mRNAs in HT-29 and caco-2 cells infected with RV (0.3 MOI, 24hpi).

qRT-PCR validation of selected differentially expressed lncRNAs in HT-29 and caco-2 cells infected with RV.

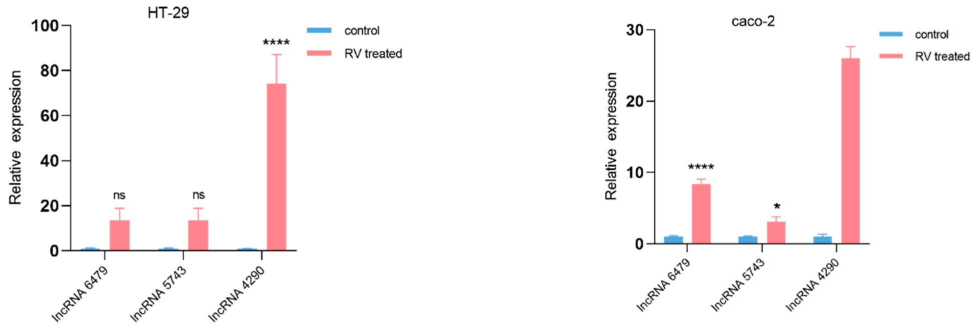

Figure S2. qRT-PCR validation of lncRNAs in HT-29 and caco-2 cells infected with RV (0.3 MOI, 24hpi).

Effect of C3 and OASL inhibition was detected by western blotting.

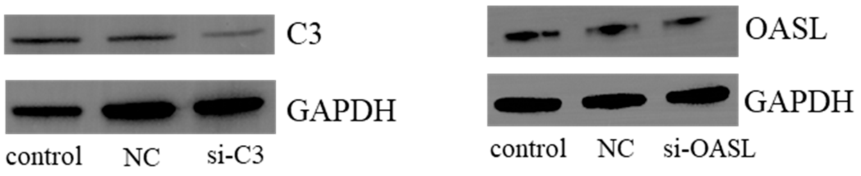

Figure S3. the siRNA experiment of C3 and OASL.

Effect of lncRNA 6479 and lncRNA 4290 inhibition was detected by RT-PCR.

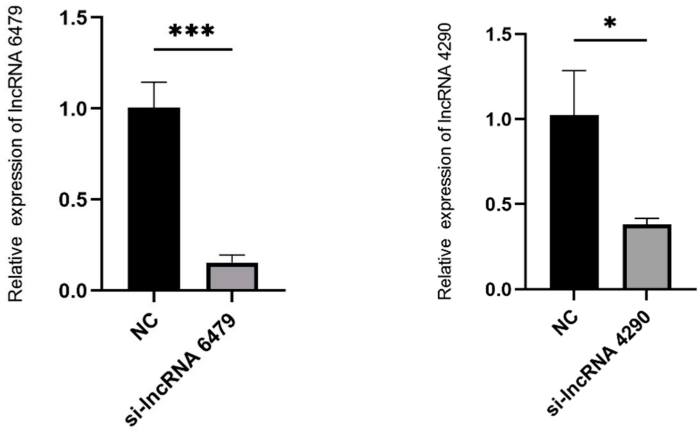

Figure S4. the siRNA experiment of lncRNA 6479 and lncRNA4290.

Effect of C3, OASL, lncRNA 6479 and lncRNA 4290 inhibition on RV replication at 16 hpi in MA104 cells, examined using RT-PCR.

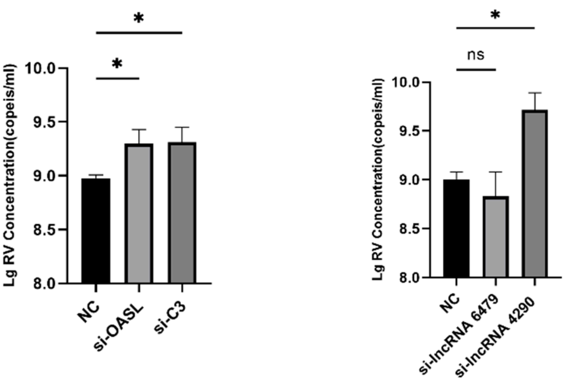

Figure S5. the effect of C3, OASL and lncRNA 6479 inhibition on RV replication (0.1 MOI, 16 hpi after transfection for 48h)
